# P-467. The clinical prognostic risk stratification system for HIV infected hepatocellular carcinoma

**DOI:** 10.1093/ofid/ofae631.666

**Published:** 2025-01-29

**Authors:** Yifan Chen, Han Zhao, Yao Wang, Bo Liu, Zhimin Chen, Yu Tao, Yang Xun, Hua Yang, Rongqiu Liu, Lizhi Feng, Xinhua Liu, Hengjing Li, Sibo Wang, Dong Zhao, Haolan He, Hua You

**Affiliations:** Children's Hospital of Chongqing Medical University, Chongqing, Chongqing, China; Guangzhou Eighth People's Hospital, Guangzhou, Chongqing, China; Affiliated Cancer Hospital & Institute of Guangzhou Medical University, Guangzhou, Guangdong, China; Guangzhou Eighth People's Hospital, Guangzhou, Chongqing, China; Guangzhou Eighth People's Hospital, Guangzhou, Chongqing, China; Children's Hospital of Chongqing Medical University, Chongqing, Chongqing, China; Foshan University, Foshan, Guangdong, China; Foshan University, Foshan, Guangdong, China; Children's Hospital of Chongqing Medical University, Chongqing, Chongqing, Chongqing, China; Guangzhou Eighth People's Hospital, Guangzhou, Chongqing, China; Guangzhou Eighth People's Hospital, Guangzhou, Chongqing, China; Guangzhou Eighth People's Hospital, Guangzhou, Chongqing, China; Guangzhou Eighth People's Hospital, Guangzhou, Chongqing, China; Shenzhen Third People’s Hospital, Shenzhen, Guangdong, China; Guangzhou Eighth People's Hospital, Guangzhou, Chongqing, China; Children's Hospital of Chongqing Medical University, Chongqing, Chongqing, China

## Abstract

**Background:**

Patients with human immunodeficiency virus (HIV) are more susceptible to liver cancer because of their compromised immune system. There is no specific prognostic model for HIV-infected hepatocellular carcinoma (HCC) patients.

**Methods:**

Clinical data of 85 patients with HIV-infected HCC was divided into a 7:3 ratio for training and internal validation sets, while the data of 23 patients with HIV-infected HCC was served as the external validation set. Data of 275 HIV-negative HCC patients was considered as external HIV-negative validation set. Variables associated with overall survival (OS) in the training set were used to develop the HIV-infected HCC prognosis (HIHP) model. The model was tested in the internal and external validation sets. The predictive accuracy of the model was assessed with conventional HIV-negative HCC prognostic scoring systems.

**Results:**

The HIHP model demonstrated a significant association with OS in the training set, with a median OS of 13 months for low risk, 7 months for medium risk, and 3 months for high risk (*p* < 0.001). The HIHP model showed a significant association with OS, and exhibited greater discriminative abilities compared to conventional HIV-negative HCC prognostic models both in the internal and external validation sets. In the external HIV-negative validation set, the HIHP model did not show better discrimination than conventional HIV-negative HCC scores.

**Conclusion:**

The new model presented in the work provided a more accurate prognostic prediction of OS in HIV-infected HCC patients. However, the model is not applicable to patients with HIV-negative HCC.

**Disclosures:**

**All Authors**: No reported disclosures

